# Source Credibility and the Information Quality Matter in Public Engagement on Social Networking Sites During the COVID-19 Crisis

**DOI:** 10.3389/fpsyg.2022.882705

**Published:** 2022-06-16

**Authors:** Zakir Shah, Lu Wei

**Affiliations:** College of Media and International Culture, Zhejiang University, Hangzhou, China

**Keywords:** elaboration likelihood model (ELM), perceived benefit, perceived risk, public engagement, social networking site

## Abstract

During the coronavirus disease 2019 (COVID-19) pandemic, people use social networking sites (SNSs) to seek social support, ease the move toward the social distance, and communicate and engage with one another. However, there is growing evidence that trustworthiness and quality of information can affect individuals’ online engagement behaviors. This study proposes a theoretical model to test people’s online engagement during the COVID-19 pandemic by applying the elaboration likelihood model (ELM). Through a questionnaire survey of 630 SNS users, the study examines whether and how source credibility and information quality affect people’s online engagement during the COVID-19 pandemic. The model was tested using structural equation modeling. The findings show that source credibility and information quality have a significantly positive relationship with perceived benefit, while negative and significantly associated with perceived risk. Furthermore, perceived benefit is a stronger predictor of online public engagement than the perceived risk. To improve online public engagement as a crisis response strategy, careful source selection and careful generation of online crisis information should not be overlooked.

## Introduction

In the last month of the year 2019, a new type of disease, officially named by WHO as coronavirus disease 2019 (COVID-19), was reported by the Chinese health authorities, raising momentous concerns among the global health experts and governments (Simione and Gnagnarella, 2020; Hassan et al., 2022). This mystery disease, now known to be caused by the new type of virus identified as “2019 novel coronavirus” (COVID-19), was first reported in the populous city of Wuhan in Hubei Province, China, and is declared to be of zoonotic (an infection that comes from animals) origin (Li et al., 2020; Holmes et al., 2021). COVID-19 causes serious illness, having symptoms of common cold and pneumonia, which is appearing to be more deadly and contagious than the previously known similar infections (severe acute respiratory syndrome and Middle East respiratory syndrome). As of October 20, 2021, the coronavirus has affected more than 240 million people while the number of deaths has crossed the figure of four million globally (Khan et al., 2022). Research indicates that as a result of COVID-19 people all over the world have eased the move to social distance by spending a lot of time online. Social networking sites (SNSs) have seen a 61% rise in use as individuals utilize sites to search or share information and maintain engagement with families, colleagues, and friends (Nabity-Grover et al., 2020). Individuals compensate for decreased accessibility to their conventional social support by using a variety of Internet-based communication technologies to communicate and interact with others. Adopting certain technologies was crucial to minimize potential depressive episodes and growing levels of anxiety arising with unexpected restrictions as to how people engage with one another (Harrison and Johnson, 2019; Nabity-Grover et al., 2020).

During emergencies and disasters, SNSs became powerful communication and public engagement platforms (Hong and Kim, 2019; Shah et al., 2019). Practitioners and institutional managers use SNSs to deliver information, build social trust and capital, and encourage people’s engagement in recuperative activities. The public, however, often uses SNSs, a new and widely used way of communication and engagement (Shah et al., 2019). The term “engagement” refers to a “psychologically motivated effective state that leads to extra-role behaviors” (Kang, 2014, p. 402). In other words, engagement refers to how people react to social media material and what they do with it (Smith and Derville, 2015). Similarly, engagement on SNSs indicates users’ attitudinal and behavioral responses to online content, such as searching, posting, and liking (Tsai and Men, 2018); demonstrating support and criticism; and exchanging knowledge with mutual connections (Men and Tsai, 2013). In this study, public engagement on SNSs is described as the intentional behaviors of people during crises, for using SNSs to obtain and provide information and social support, and to involve in negative word of mouth (Zhang et al., 2018).

Individuals are creating social capital through online engagement, enhancing their self-worth, and self-esteem, and fulfilling their pleasure needs (Heravi et al., 2018). Similarly, the perceived social and enjoyment benefits have a positive impact on online public engagement (Jozani et al., 2020). In contrast, growing connectivity and engagement through SNSs have contributed to an increased risk of violation of privacy (Watson and Rodrigues, 2018; Jozani et al., 2020). Government agencies, as well as the public, may face challenges in online public engagement such as the digital divide, trustworthiness and transparency, privacy and security concerns, and information risk (Harrison and Johnson, 2019; Chen et al., 2020), which ultimately reduces people’s online engagement. Therefore, communication experts need to understand how perceived benefits and risks can influence online public engagement during a crisis.

Previous research revealed that during a crisis people are more likely to receive unverified or inaccurate information on SNSs, which can cause uncertainty and have negative health consequences (Shim and Jo, 2020). Similarly, the ever-increasing availability of information, as well as the proliferation of numerous online platforms and applications, may result in information overload (Fu et al., 2020; Shim and Jo, 2020). Individuals, in contrast, do not trust or engage with SNSs as a result of information overload or low-value information such as spam and fake news (Liu et al., 2020; Apuke and Omar, 2021). Moreover, high-quality visual contents have unique attributes and features that impact the level of engagement of individuals on SNSs (Cao et al., 2021). For example, the structure, content, and style of messages during the Boston Marathon Bombing disaster significantly influenced people’s online information-sharing behaviors (Sutton et al., 2015; Liu et al., 2020). Therefore, one may wonder how a person’s trust in and the credibility of the source and information quality may affect his/her online engagement behaviors, particularly during crises.

**FIGURE 1:**
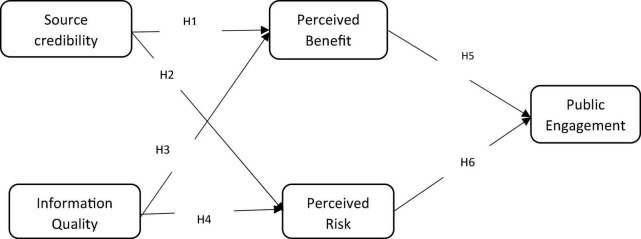
Proposed theoretical framework.

Research indicated that communication through effective communication channels and media develops public trust in organizations or sources, which in turn promote public engagement (Flora Hung-Baesecke et al., 2016). Similarly, the credibility of media and sources influences the media attitudes of individuals that inevitably influence their perception of media trust and credibility (Vander Molen et al., 2015; Visentin et al., 2019). In addition, the perceived credibility of the source influences individuals’ engagement behaviors on SNSs (Men and Tsai, 2013). Past research has described various internal and external factors such as perceived social support, enjoyment, interactivity, and privacy risk, and information risk that influence people’s online engagement intentions during a crisis (Jiang et al., 2016; Molinillo et al., 2019; Shah et al., 2019; Cheng and Jiang, 2020). Relatively few researchers have examined the impact of situational factors such as trustworthiness, media richness, the credibility of the source, and the quality of information connected to public engagement *via* SNSs during a crisis. Thus, this study proposed a conceptual model (see Figure 1) by referring to the elaboration likelihood model (ELM) and public engagement on SNSs.

This study makes some contributions to the literature. First, the study aims to identify the factors that may affect the motives behind the public engagement on SNSs during a crisis. In particular, the study examined how the processing of information *via* the dual route (source credibility as peripheral route and information quality as central route) influences individuals’ perception (perceived benefits and perceived risk) of online engagement during COVID-19. Second, previous research has applied the ELM in a variety of contexts, but there is a lack of research in the context of crisis communication, particularly based on COVID-19. As a result, this is one of the few studies that expanded the ELM theory in the context of crisis communication, with a focus on the COVID-19 pandemic.

## Theoretical Background and Hypothesis Building

### Public Engagement

In recent times, public engagement has taken on a new momentum with the advent of social media outlets such as Twitter, YouTube, and Facebook (Golbeck et al., 2010), all of which have the shared function of facilitating real-time interaction. Encouraging civic engagement, interactivity, and real-time communication of this nature is potentially useful since it becomes easier for people to participate in public life. Although the number of public-sector studies on social media is increasing (e.g., Lee and Kwak, 2012; Smith and Derville, 2015), there has been less evidence to date on the role of social media in fostering public engagement and, more specifically, on the two modes of public communication and public involvement (Agostino and Arnaboldi, 2016). Public engagement is generally characterized as the participation of people in public relations (Huang et al., 2021), and its role is to create a partnership between organizations and their people that goes beyond simply exchanging information.

Crises are unforeseen and non-regular occurrences that bring corporations and its publics at risk and disruptions (Zhang et al., 2018). Crisis literature recently discussed the critical roles of crisis communication in mitigating people’s perception of threats and confusion and also encourages the public to restore confidence and deal with psychological stress (Jin et al., 2012; Coombs and Holladay, 2014). The public relies on SNSs for informational and social support as a means of competing with the crisis (Shah et al., 2019). However, there may be a considerable gap in determining the relationship between individuals’ processes and their engagement with SNSs, which needs further exploration (Grappi et al., 2013). In particular, there is still a need for development of an understanding of how personality attributes, including psychological, cultural, or emotional behaviors, contribute to public engagement *via* SNSs. Effective use of SNSs in times of crisis requires an understanding of the motives for public engagement on SNSs. Therefore, this study aims to investigate how information quality, source credibility, perceived benefits, and perceived risk influence individuals’ online engagement behaviors during a crisis.

### Elaboration Likelihood Model

Petty and Cacioppo (1986) developed the ELM by using a mechanism of the dual-process model to investigate attitude development, influences, or changes in behaviors. Elaboration was defined by the ELM as the extent to which individuals think of the arguments embedded in the message (information). Previous research classified the factors that influence the amount of elaboration based on individuals’ motivation and ability (O’Keefe, 2013). Similarly, individuals’ motivation for elaboration is influenced by their perceived personal relevance, the degree of need for cognition, the source of the message, and the importance of the issue. While intelligence, time available, message repetitiveness, complexity, relevant experience, level of knowledge, and the amount of distraction in the message environment all have an impact on an individual’s ability to elaborate a message (Goh and Chi, 2017). Furthermore, the level of motivation, ability, preoccupation, and belief of individuals determines which route will be taken to elaborate the message contents (Kitchen et al., 2014; Yoo et al., 2020).

The ELM model indicates that the attitudes of individuals are transformed by two separate paths, namely, peripheral and central ways (Zha et al., 2018). In the central path, people critically consider the issues related to the arguments raised in the information message, including the content, text, and language of the message, under conditions of high elaboration (Ott et al., 2016). Therefore, the central path requires a lot of cognitive effort to comprehend a message. In contrast, the peripheral approach needs comparatively less cognitive effort as individuals rely on heuristic signals such as the credibility of the source, reputation, attractiveness, and popularity to persuade individuals and influence their attitudes and intentions (Kang and Namkung, 2019; Xu and Warkentin, 2020).

Li (2013) introduced ELM as a referent theory for information system acceptance and elaborates on different types of persuasive messages (source credibility and arguments quality) on social influence, and individuals’ cognitive and affective responses. Similarly, scholars applied ELM to social-mediated communication contexts that examined how corporates’ posts’ functional traits and emotional features of information predict public engagement on social media (Chun and Lee, 2016; Ott et al., 2016; Ji et al., 2017; Cyr et al., 2018). Previous studies on ELM in online environments have identified several features, including the availability of interactive features, navigation design, and connectedness (Goh and Chi, 2017; Cyr et al., 2018).

In previous studies, the ELM has been applied to online marketing (Cyr et al., 2018), customers’ initial trust in mobile banking (Gu et al., 2017), crowdfunding intentions (Wang and Yang, 2019), and other contexts, including mHealth user acceptance, and privacy context in mHealth (Zhu et al., 2021). However, the research described the information quality and source credibility as factors that enable individuals to acknowledge the information on SNSs effectively (Tseng and Wang, 2016; Kang and Namkung, 2019). Factors that drive social media engagement are generally related to the post’s creator, the post’s context, and the features of the content (Il Shin et al., 2021). Thus, this research extends the range of the ELM and verifies the validity of the dual route of the ELM in the context of online public engagement during a crises situation. The study examined how the processing of information *via* the dual route (source credibility as peripheral route and information quality as central route) influences individuals’ perception (perceived benefits and perceived risk) of online engagement during COVID-19.

### Credibility of Source

The credibility, attractiveness, and competence of the source have great importance in the usage of SNSs, particularly in the area of crisis communication. During a crisis, people tend to the online environment to search or provide crisis information (Balouchi et al., 2018; Shah et al., 2019; Weismueller et al., 2020). However, despite the impressive role of SNSs during a crisis, users are still worried about the credibility of information and its anonymity contributors (Liao and Mak, 2019). The source’s credibility of a message is essential for the public as it reflects the trustworthiness of an individual and group who creates a message (Yin and Zhang, 2020). Prior studies show that the qualities of a credible source such as attractiveness, trustworthiness, and expertise have a significant persuasion effect on people’s beliefs, attitudes, and behaviors (Ryu et al., 2018; Sokolova and Kefi, 2020). In addition, the structure of a message, demographic factors of sender and receivers, and their experience can influence people’s perception regarding the credibility of a source (Weibel et al., 2008).

Previous research shows that source credibility has a positive effect on individuals’ perceived benefits (i.e., perceived usefulness and perceived ease of use) in the acceptance of technology systems (Li, 2013), and experience in online purchasing (Kang and Namkung, 2019). In contrast, Sun and Meng (2017) found that source credibility *via* SNSs can affect people’s perceived risk and intentions to purchase genetically modified foods. Furthermore, Ryu et al. (2018) found an indirect effect of source credibility on people’s perceived risk and acceptance of nuclear power. Past research has demonstrated a significant correlation between the credibility of sources and individuals’ benefits and risk in the field of online purchasing behaviors (Clow et al., 2008), tourists’ behaviors (Balouchi et al., 2018), and climate information on media and individuals’ risk perception (Dong et al., 2018). However, there is a lack of research in the field of crisis and health communication that how source credibility of information may affect the perceived risk and benefits of online public engagement in a crisis like during the COVID-19 pandemic. Thus, this study suggests the following hypotheses:

**H1: Source credibility has a negative relationship with the perceived risk of online public engagement during the COVID-19 crisis.**

**H2: Source credibility has a positive correlation with the perceived benefits of online public engagement during the COVID-19 crisis.**

### Information Quality

Scholars describe information quality as “consumers’ perception about the information based on a collection of decision criteria that cover accuracy, validity, usefulness, up-to-datedness and impartiality” (Ou and Sia, 2010). In the literature on individuals’ behaviors, information quality, virtual interactivity, and incentives have a strong and positive effect on online public engagement (Islam and Rahman, 2017). High-quality information in online communities offers users a wealth of knowledge that strengthens their positive attitudes toward online engagement (Dessart et al., 2015). Similarly, Shim and Jo (2020) found that the quality of information had a positive correlation with the perceived benefits and satisfaction of users and the intention to reuse sites in health settings. Furthermore, information quality influences users’ satisfaction (Ghasemaghaei and Hassanein, 2015) and brand equity (Barreda et al., 2015; Kang and Namkung, 2019). In addition, perceived benefits such as perceived ease of use, perceived usefulness, and satisfaction affect the customer attitudes and behaviors to brand engagement *via* the SNSs (Hollebeek et al., 2014; Hsieh and Chang, 2016). When the information is well structured and has high quality (more relevant, accurate, and comprehensive), it may reduce people’s risk perception and uncertainty associated with acting on information (Yi et al., 2013). Therefore, in this study, we suggested the following hypothesis:

**H3: Information quality has a positive relationship with the perceived benefits of online public engagement during the COVID-19 crisis.**

In contrast, if the quickest-growing amount of available information and accumulation of numerous online platforms and applications are followed by an inadequate health awareness and knowledge, it could result in an unforeseen by-product – information overload (Jensen et al., 2017). When people are overloaded by online information, and especially if the quality of the information is uncertain, it will be tough for them to fully understand it (Jiang and Beaudoin, 2016). In addition, people could be confused and make hasty and ill-informed decisions that may have unforeseen, adverse health effects (Islam and Rahman, 2017; Shim and Jo, 2020). Living in a fast technological era and the rise of SNSs, communication experts and other practitioners provide diverse and sufficient information on SNSs to meet individual crisis information needs. Therefore, individuals with high perceived risk require more convincing arguments such as high-quality information to reduce their risk perception. High-quality information, such as reliable, timely, appropriate, and detailed, is more likely to reduce the perceived risk of individuals (Tseng and Wang, 2016), and also can enhance online public engagement during a crisis. Therefore, this study suggests the following hypothesis:

**H4: Information quality has a negative relationship with the perceived risk of online public engagement during the COVID-19 crisis.**

### Perceived Risk

Perceived risk is a critical element of individuals’ behaviors as the study mainly identifies this component as an alleged confusion about the outcomes and the consequences of using SNSs (Lee and Song, 2013; Tseng and Wang, 2016). Perceived risk refers to individuals’ awareness and assessment of the insecurity and repercussion as a result of their decision-making (Joo et al., 2021). This study describes perceived risk as the potential users’ risk about the possible undefined negative consequences of online engagement during a crisis. Unfavorable ethical views of social media analytics procedures and poor trustworthiness contribute to the perceived risk of information sharing (Hajli and Lin, 2016; Le and Liaw, 2017). For example, the collection and usage of consumers’ information for business purposes is an invasion of their privacy and leads to uncertainty and concerns regarding the outcomes of disclosing online information (Hajli and Lin, 2016; Le and Liaw, 2017). Research reveals that perceived risk associated with online engagement will reduce individuals’ concepts of behavioral and environmental regulation that can negatively affect their decision to use SNSs (Lorenzo-Romero et al., 2011). In addition, lack of trust in source and perceived risks mitigate the perceived benefits and intentions to use and share information on SNSs (Lorenzo-Romero et al., 2011; Punj, 2017). Moreover, perceived risk (institutional and social privacy risks) reduces individuals’ online engagement (Jozani et al., 2020). Past work has investigated the potential risks of using SNSs; however, they have primarily focused on its initial adoption and usage, while the long-term behavioral effects are ignored. Furthermore, there is a lack of research that examines how perceived risk influences public engagement on SNSs in a crisis. Thus, we proposed the following hypothesis:

**H5: Perceived risk has a negative relationship with public engagement on SNSs during the COVID-19 crisis**.

### Perceived Benefits

Previous research indicated that perceived benefits (social interaction and enjoyment benefits) have a positive effect on individuals’ engagement behaviors (Jozani et al., 2020). Perceived enjoyment and satisfaction push users far beyond fundamental hedonic values (Pang, 2021), whereby using the provider site, the user feels emotional engagement (Mirzaei and Esmaeilzadeh, 2021). Research has found that a website’s features such as enjoyable, interesting, exciting, and entertaining enjoyment has a positive impact on users’ assessment of a website (Ou and Sia, 2010; Jozani et al., 2020). Some other scholars comprehend the ability of virtual reality in engaging and influencing individuals’ behaviors and suggest that virtual reality features may offer both utilitarian and hedonic benefits to consumers (Rauschnabel et al., 2018; McLean and Wilson, 2019). Similarly, perceived benefits such as perceived ease of use, perceived usefulness, and satisfaction affect the customer attitudes and behaviors to brand engagement *via* SNSs (Arghashi and Yuksel, 2021). Past research indicates that the perceived quality of information, perceived enjoyment, and interactivity are acceptable factors that can influence public engagement in SNSs (Reitz and Yan, 2012). Therefore, this study assumes the following hypothesis:

**H6: Perceived benefit has a positive association with users’ online engagement during the COVID-19 crisis.**

## Methodology

### Data Collection and Analysis

In this study, a cross-sectional online survey was conducted during the period from 1 February 2020 to 30 March 2020, the early phase of the COVID-19 outbreak in China. The data were collected from a large university in Mainland China. All the participants are students and research scholars; therefore, the number of young participants is high in the study. A questionnaire was sent through WeChat to various groups and personal contacts. The respondents were assured that their data would be kept anonymous. A total of 730 participants were invited, and we received responses from 670 (92%). Out of 670, 40 responses were discarded due to inadequate replies. Finally, we considered 630 responses for further analysis. After the data collection, the demographic information of respondents was found *via* descriptive analysis. The sample consisted of 353 (56%) men and 277 (44%) women (for more details, see Table 1). Next, the exploratory factor analysis was performed to test the reliability and validity of the variables. Finally, we used AMOS 21 with structural equation modeling’s (SEM’s) comprehensive techniques to define the confirmatory factor analysis (CFA) and model fit indices of the current theoretical framework.

**TABLE 1:** Demographic of respondents.

Category		Frequency	%
Gender	Male Female	353 277	56 44
Age	20–25 Years 25–30 years Above 30	208 302 120	33 48 19
Education	Master Ph.D. Postdoc	189 391 50	30 62 8
Experience with SNSs use	0–3 years 4–5 years Above 6 years	195 280 155	31 44 25

### Measurements

To test our proposed research model, we develop a questionnaire and modified the items from the past research. A five-point Likert scale from 1 = strongly disagree to 5 = strongly agree was used to measure items of the constructs.

### Information Quality

To measure the information quality, we adapted a three-item scale from Zha et al. (2018). The items indicate individuals’ perceptions about the currency, accuracy, and completeness of information on SNSs. Example items include (i) COVID-19-related information on SNSs is accurate; (ii) COVID-19-related information on SNSs is sufficient and timely; and (iii) COVID-19-related information on SNSs is comprehensive. Cronbach’s alpha (CA) value is 0.98.

### Source Credibility

Source credibility was tested with three items that were adapted from Zha et al. (2018). The items defined the view of respondents about the trustworthiness, expertise, and credibility of the source of information on SNSs. Example items include (i) the person who posted COVID-19-related information on SNSs is trustworthy; (ii) the person who posted COVID-19-related information on SNSs is knowledgeable; and (iii) the person who posted COVID-19-related information on SNSs is an expert. CA value is 0.84.

### Perceived Benefits

Perceived benefits of engagement *via* SNSs were tested using a three-item scale that was adapted from Hussain et al. (2017). Example items include (i) COVID-19-related information on SNSs is informative; (ii) COVID-19-related information on SNSs is valuable; and (iii) COVID-19-related information on SNSs is helpful. CA value of the scale is 0.87.

### Perceived Risks

To measure individuals’ perceived risk of engagement on SNSs, we adapted a two-item scale from Shah et al. (2019). The items indicate individuals’ risk perception regarding the usage of SNSs during a crisis. Example items include (i) I think I should avoid public engagement on SNSs during the COVID-19 to secure my privacy, time, and money and (ii) I think engagement on SNSs during the COVID-19 crisis is riskier than the expected benefits. The observed value of CA is 0.95.

### Public Engagement

Public engagement was tested by using a four-item scale that was adapted from Shah et al. (2019). Example items include (i) I watch videos and read messages/posts and users’ comments on SNSs to stay informed during the COVID-19 crisis; (ii) I like, comment, and share information on SNSs to help people during the COVID-19 crisis; (iii) I exchange information related to COVID-19 crisis on SNSs to seek or provide help to others in the decision-making process; and (iv) I upload information, videos, and other graphical contents related to COVID-19 crisis on SNSs. CA value is 0.88.

### Reliability and Validity

Cronbach’s alpha, factor loadings (FLs), composite reliability (CR), and average variance extracted (AVE) were used to assess constructs’ reliability and validity. Table 2 shows the results of CA, FL, CR, and AVE, which indicates that all the values of CA, FL, CR, and AVE are in the acceptable range recommended by Hair et al. (2014). Furthermore, Table 3 shows that all values (in bold) of the square root of AVEs are greater than the intercorrelations of the variables, implying discriminant validity.

**TABLE 2:** Reliability and validity.

Construct		Loadings	Cronbach’s alpha	AVE	CR
	IQ1	0.972			
Information Quality (Zha et al., 2018)	IQ2	0.974	0.98	0.95	0.98
	IQ3	0.971			
	SC1	0.846			
Source Credibility (Zha et al., 2018)	SC2	0.838	0.84	0.73	0.89
	SC3	0.881			
	PB1	0.870			
Perceived benefits (PB) (Hussain et al., 2017)	PB2	0.907	0.87	0.77	0.91
	PB3	0.862			
Perceived risks (PR) (Shah et al., 2019)	PR1	0.967	0.95	0.93	0.96
	PR2	0.962			
	PE1	0.806			
Public engagement (PE) (Shah et al., 2019)	PE2	0.849	0.88	0.73	0.91
	PE3	0.878			
	PE4	0.877			

*AVE, average variance extracted; CR, composite reliability.*

**TABLE 3:** Mean, standard deviation, and correlation.

	Mean	*SD*	1	2	3	4	5	6	7	8	9
(1) Gender	1.44	0.497									
(2) Age	1.86	0.708	0.190**								
(3) Education	1.78	0.576	−0.240**	0.342**							
(4) Experience	1.94	0.743.	0.008	−0.225**	0.016						
(5) IQ	3.83	1.11	0.164**	0.021	0.027	0.224**	**(0.974)**				
(6) SC	4.08	0.628	0.081*	−0.167**	−0.169**	0.278**	0.131**	**(0.854)**			
(7) PB	3.66	0.867	−0.082*	0.004	–0.006	0.260**	0.212**	0.207**	**(0.877)**		
(8) PR	3.48	1.121	0.221**	0.116**	0.196**	−0.151**	−0.142**	−0.206**	−0.133**	**(0.964)**	
(9) PE	3.88	0.824	0.015	–0.050	–0.054	0.176**	0.135**	0.228**	0.251**	−0.097*	**(0.854)**

**p < 0.01, *p < 0.05. n.s, non-significant; IQ, information quality; SC, source credibility; PB, perceived benefits; PR, perceived risks; PE, public engagement.

*Bold values stands for discriminant validity.*

Furthermore, the common method bias (CMB) analysis was carried out using the methods proposed by Podsakoff et al. (2003). We perform the Harman single-factor analysis (Harman, 1976), and the results show that 28.11% of the variance is explained by a single factor, which is less than the cutoff value of 50%, indicating that CMB is not a problem.

In addition, using AMOS-21, CFA was used to check the model fitness indices of the measurement model. The results are within a widely accepted range. The root mean square error (RMSEA) is 0.041, which is less than the 0.10 threshold value (Anderson et al., 1988). χ^2^/df is 2.078, which falls within the recognized range as well. In addition, goodness-of-fit index (GFI) is 0.982, the adjusted goodness of fit (AGFI) is 0.95, normed fit index (NFI) and the tucker-lewis index (TLI) are 0.98 each, and the comparative fit index (CFI) is 0.99. All values are above the 0.90 estimates and were explained by Hair et al. (2010). The results show a good measurement model fit.

### Structural Model

Previous researchers have used SEM to evaluate their theoretical frameworks. It is a multivariate technique that incorporates multiple regression aspects and factor analysis to test a set of complex relations of dependency at the same time (Hair et al., 2010). Anderson et al. (1988) described that if the value of χ^2^/df is between the range of 0 and 3 it is considered acceptable. For this study, the χ^2^/df value is 2.24, which is in the acceptable range. Similarly, the value for RMSEA is 0.044, which is less than the recommended value of 0.08. Furthermore, the values are as follows: GFI 0.96, AGFI 0.94, NFI 0.97, TLI 0.98, and CFI 0.98; All these values are above the benchmark value of 0.90 (Hair et al., 2010), which means considerably acceptable. Thus, the findings demonstrate a valid model fit.

**FIGURE 2:**
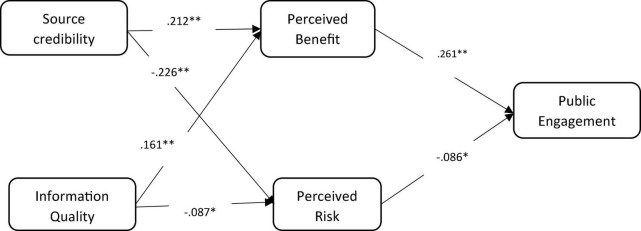
Proposed framework with β values. **P < 0.01, *P < 0.05.

After the fitness of the model, the association between the two exogenous sources of credibility and information quality, and three endogenous constructs, including perceived benefits and risks, and public engagement on SNSs, was assessed. As shown in Figure 2, in the peripheral route, the effect of source credibility of COVID-19-related information is significantly and positively associated with perceived benefits with the magnitude of β = 0.212, *p* < 0.01, thus H1 is supported. Similarly, source credibility is significantly and negatively associated with perceived risk with a magnitude of β = –0.226, *p* < 0.01, leading to accepting H2.

Regarding the central route, the effects of information quality on perceived benefits were significant and positive as statistically described as β = 0.161, *p* < 0.01, thus accepting H3. While information quality is significantly and negatively associated with perceived risks with a magnitude of (β = –0.087, *p* < 0.05), thus H4 is supported. In addition, perceived benefit and perceived risk are significantly associated with public engagement as statistically described as β = 0.261, *p* < 0.01, and β = –0.086, *p* < 0.05, respectively; therefore, H5 and H6 are accepted. The results found that source credibility and information quality are the main factors that reduce the perceived risks and boost the perceived benefits of using SNSs during a crisis. Whenever people have high perceived benefits and low levels of risk, it leads to public engagement on SNSs during crises. However, the relationship between perceived benefits with public engagement is stronger than the negative relationship between perceived risk with public engagement. It means that high quality of information and source credibility reduce individuals’ perceived risk of individuals, which in turn influences their intentions of seeking, sharing, commenting, or liking information and content on SNSs during a crisis.

## Discussion

This study examines how source credibility and information quality influenced public engagement on SNSs during the COVID-19 crisis. The findings show that source credibility and information quality are significantly and positively associated with the perceived benefits of public engagement on SNSs while having a significant negative relationship with the perceived risk of public engagement on SNSs. Moreover, perceived benefit is significantly associated with public engagement. When individuals enjoy communicating with others, they are more likely to receive more information, increase their knowledge, and build social capital through these interactions (Wang and Liu, 2019). Similarly, perceived risk has a negative association with public engagement on SNSs. However, the interaction between perceived benefit and public engagement was stronger than that between perceived risk and public engagement.

In the first hypothesis, the findings show that source credibility has a significant positive relationship with the perceived benefits of individuals’ engagement with SNSs. The findings are consistent with previous studies (Aderonke, 2010; Li, 2013; Kang and Namkung, 2019), where they found that source credibility has a positive impact on individuals’ perceived usefulness and perceived ease of use, in the acceptance of technology and online purchasing environment. Similarly, in response to H2, the findings show that source credibility has a significant negative association with the perceived risk of online engagement during the COVID-19 crisis. The findings support the previous research of Balouchi et al. (2018) and Zhang and Yang (2019) who found that source credibility hurts the perceived risks of the review of disabled guests and tourists. They found that source credibility can significantly reduce the risk perception of disabled guests (Zhang and Yang, 2019).

The findings of H3 reveal that information quality has a significant positive relationship with the perceived benefits of online public engagement during the COVID-19 crisis. The analysis supports previous studies that showed that information quality had a positive association with the perceived benefits and satisfaction of individuals and intentions to reuse the information sites and brand (Islam and Rahman, 2017; Shim and Jo, 2020). Similarly, in response to H4, the findings show that information quality affects negatively the perceived risk of public engagement on SNSs during COVID-19. This further supports previous literature that claimed that information with high quality and convincing arguments can reduce the risk perception of people (Tseng and Wang, 2016). Similarly, Shim and Jo (2020) described that unverified, wrong, or conflicting information on SNS can have adverse health effects on individuals.

The findings of this study demonstrate that the peripheral approach has a greater impact on individuals’ perception and engagement behaviors than the central path. The possible reason for this may be that the peripheral approach needs comparatively less cognitive effort as individuals rely on heuristic signals such as the credibility of the source, reputation, attractiveness, and popularity to persuade individuals and influence their attitudes and intentions (Kang and Namkung, 2019; Xu and Warkentin, 2020). In contrast, the central path requires a lot of cognitive effort to comprehend a message.

Regarding H5, the results reveal that perceived risks affect negatively public engagement on SNSs. The result is consistent with Hajli and Lin’s (2016) and Jozani et al.’s (2020) findings that institutional and social privacy risks mitigate a person’s online engagement. In addition, perceived risk reduces the perceived benefits of SNS use that ultimately can negatively influence individuals’ intentions to use SNSs (Lorenzo-Romero et al., 2011). However, the findings of H6 suggested that perceived benefits have a significant positive correlation with public engagement on SNS during the COVID-19 crisis. The findings support Hsieh and Chang’s (2016) and Jozani et al.’s (2020) findings where they found that perceived benefits such as perceived usefulness, satisfaction, social capital, and enjoyment have a positive effect on people’s engagement behaviors. Moreover, perceived utilitarian and hedonic benefits positively influence consumers’ engagement behaviors (McLean and Wilson, 2019). Overall, the perceived benefit has a greater impact on individuals’ engagement behaviors, implying that online engagement during crises is generally beneficial in terms of improving individuals’ connectivity, satisfaction, awareness, and knowledge. As a result, online engagement is more beneficial to individuals during a crisis than the perceived risks associated with it.

### Theoretical Implications

While communication scholars continue to adapt to a more stakeholder-centric model of online public engagement during the crisis, research is still in the process of better understanding the mechanism behind public engagement on SNSs during a crisis. The findings of this study offer an enhanced understanding of public engagement on SNSs during the COVID-19 crisis and move forward in this growing area of research. Incorporating a theoretical perspective from the ELM, this research adds to crisis communication literature by investigating how people cognitively and effectively assess the contextual factors of crisis information and involvement in public engagement on SNSs during a crisis. First, this study clarifies the role of source credibility and information quality in the process of public engagement during a crisis. The findings show that source credibility and information quality are significantly and negatively linked with the perceived risks while significantly and positively correlated with perceived benefits of public engagement on SNSs. The findings show that credibility, expertise, trustworthiness, and high quality of information play a crucial role in influencing the perception and engagement behaviors of people. Second, the study investigates why individuals engage in SNSs despite the potential perceived risks. The findings show that both perceived benefits and risks affect people’s online engagement behavior. However, the association between perceived benefits and public engagement was stronger than that of the perceived risks. Therefore, the study indicates that despite the potential perceived risks of using SNSs, the consideration of perceived benefits positively influences individuals’ online engagement behaviors.

### Managerial Implications

This study also offers some practical implications for crisis communication practitioners. The findings show that information quality and source credibility may encourage people’s online engagement behaviors. To develop online public engagement during a crisis like the COVID-19 pandemic, public relations practitioners need to provide accurate, consistent, timely, and understandable information. Similarly, practitioners need to provide information about the overall nature of the crisis such as causes of the crisis, damages of crisis, restorative operation by government or other public sectors, and the expected outcomes of the crisis. The advancement of information technology has made it difficult to stop the spread of disinformation, and the public is confronted with an infodemic, which may hinder their effective response to the COVID-19 pandemic (Barua et al., 2020). Therefore, practitioners should encourage individuals to evaluate the quality of information and the credibility of the source on SNSs before liking, commenting on, or sharing it from their accounts, or making any decision on a health-related issue based on information obtained through SNSs. Similarly, when confronted with conflicting information in a crisis, people must process it using prior knowledge and experience, as well as the credibility of the source. Concerning the COVID-19 pandemic, individuals do not have enough prior knowledge to directly verify the quality of information. Thus, they should evaluate or seek evidence-based information about a crisis (i.e., COVID-19 pandemic) from reputable sources such as WHO, United Nations, or any other national or international organization. Furthermore, the dissemination of high-quality information from credible sources would contribute to the successful management of the current global COVID-19 pandemic. Careful source selection and generation of online crisis information should not be overlooked to achieve the most effective outcome of online public engagement as a crisis response strategy.

Moreover, to minimize the perceived risks of using SNSs during times of crisis, users can define criteria and guidelines for the use of SNSs, provide accurate and trustworthy information, and seek permission to share or comment on users’ posts. In addition, safety and maintenance requirements should be implemented by governments and related entities to improve their validity, credibility, and security. Policymakers should take certain steps to encourage online engagement of SNSs’ users by enhancing information-seeking behaviors during times of crisis, such as improving information literacy to combat fake news and encouraging SNS users and media entities to counter false information, promote journalistic integrity, link fake news with fake-checking sites, and sanction outlets that share fake stories.

### Limitations and Directions for Future Research

This study has some limitations that should be discussed in future research. First, the study used cross-sectional survey data that showed that there were limited causal relations between the variables. In the future, researchers needed to test the model through longitudinal or experimental research. Second, in this study data were collected from a large university in China which highly represents the young population and the generalization of the study to other countries should be done with caution, considering the possible cultural difference between China and other countries. However, the novel coronavirus has affected the whole world. Therefore, researchers need to test the current model in different countries and cultures to find more interesting findings. Third, this study examined source credibility and information quality as predicting variables affecting the perceived benefits and risks of online engagement. Future researchers should test other central and peripheral factors such as source expertise, source attractiveness, system quality, and service quality that may influence individuals’ perceived benefits, risks, and online engagement intentions. Fourth, this study investigates the overall effect of information quality on individuals’ perceived benefits and risks of public engagement on SNSs. However, practitioners provide information on SNSs on location and region bases which may have a different effect on people from different regions and locations. Therefore, researchers need to decompose information quality based on location and region base to find out more interesting results. Fifth, we used a quantitative approach by using a survey technique to examine people’s online engagement during a crisis. In addition, the data used in this study were self-reported, which measure the perception and behaviors of participants not their real engagement with SNSs during the COVID-19 pandemic. Researchers need to conduct an experimental study to find out more interesting results regarding online public engagement. Finally, this study was conducted during the COVID-19 pandemic. Thus, future research should be carried out as a comparative study during and out of the COVID-19 pandemic on the use of SNSs to give more interesting results.

## Data Availability Statement

The data analyzed in this study is subject to the following licenses/restrictions: the authors have collected data and have various constructs. So the authors can’t share the data due to privacy. Requests to access these datasets should be directed to ZS, zakir@mail.ustc.edu.cn.

## Author Contributions

ZS: conceptualization, data collection and analysis, manuscript writing, proofreading, and supervising. LW: research design, theory construction, and supervising. Both authors contributed to the article and approved the submitted version.

## Conflict of Interest

The authors declare that the research was conducted in the absence of any commercial or financial relationships that could be construed as a potential conflict of interest.

## Publisher’s Note

All claims expressed in this article are solely those of the authors and do not necessarily represent those of their affiliated organizations, or those of the publisher, the editors and the reviewers. Any product that may be evaluated in this article, or claim that may be made by its manufacturer, is not guaranteed or endorsed by the publisher.
