# Supplementary material for: Source Credibility and the Information Quality Matter in Public Engagement on Social Networking Sites During the COVID-19 Crisis
Source: Front Psychol. 2022 Jun 16;13:882705. doi: 10.3389/fpsyg.2022.882705 (PMC9243660; doi:10.3389/fpsyg.2022.882705)
Supplement: Supplementary file 1 [file Table_1.docx]

Appendix

| Construct |  | Items |
| --- | --- | --- |
|  | IQ1 | COVID-19 related information on SNSs is accurate. |
| Information Quality (Zha et al, 2017) | IQ2 | COVID-19 related information on SNSs is sufficient and timely. |
|  | IQ3 | COVID-19 related information on SNSs is comprehensive. |
|  | SC1 | The person posted COVID-19 related information on SNSs is trustworthy. |
| Source Credibility (Zha et al, 2017) | SC2 | The person posted COVID-19 related information on SNSs is knowledgeable |
|  | SC3 | The person posted COVID-19 related information on SNSs is an expert. |
|  | PB1 | COVID-19 related information on SNSs is informative |
| Perceived benefits (PB) (Hussain et al., 2017) | PB2 | COVID-19 related information on SNSs is valuable |
|  | PB3 | COVID-19 related information on SNSs is helpful |
| Perceived risk Shah et al., (2003) | PR1 | I think I should avoid from public engagement on SNSs during the COVID-19, to secure my privacy, time, and money. |
|  | PR2 | I think engagement on SNSs during the COVID-19 crisis is riskier than the expected benefits. |
|  | PE1 | I watch videos and read messages/posts and users’ comments on SNSs to stay informed during the COVID-19 crisis. |
| Public engagement (PE) Shah et al., (2019) | PE2 | I like, comment, and share information on SNSs to help people during COVID-19 crisis. |
|  | PE3 | I exchange information related to COVID-19 crisis on SNSs to seek or provide help to others in decision-making process. |
|  | PE4 | I upload information, videos, and other graphical contents related to COVID-19 crisis on SNSs. |
